# Progressive flexor tendon sheath infection on hand after needle puncture during surgery: a case report

**DOI:** 10.1186/s13256-022-03525-1

**Published:** 2022-10-17

**Authors:** Gregorius Batara Putra Setia Sutardi, De Is M. Rizal Chaidir, Yoyos Dias Ismiarto

**Affiliations:** 1 Resident Physician, Orthopaedic and Traumatology Department, Faculty of Medicine, Padjadjaran University, Dr. Hasan Sadikin General Hospital, Indonesia Jl. Pasteur No. 3840161, Bandung, Jawa Barat Indonesia; 2 Hand and Microsurgery Consultant of Orthopaedic and Traumatology Department, FacultyofMedicine, Padjadjaran University, Dr.HasanSadikinGeneralHospital, Bandung, Indonesia; 3 Head of Study Program of Orthopaedic and Traumatology Department, Faculty of Medicine, Padjadjaran University, Dr.HasanSadikinGeneralHospital, Bandung, Indonesia

**Keywords:** Flexor tendon sheath infections, Hand, Needle puncture, Ray amputation, Case report

## Abstract

**Introduction:**

Flexor tendon sheath infection may be due to trauma, laceration, or bites, commonly directly inoculating the sheath. Kanavel cardinal signs in flexor tendon sheath infection cases consist of symmetrical swelling of the entire digit, a digit with semi-flexed posture, exquisite tenderness along the course of the tendon sheath, and pain with attempted passive extension of the digit. Elevated levels of inflammation markers such as white blood cell count, erythrocyte sedimentation rate, and C-reactive protein are often found in such cases. Flexor tendon sheath infections require immediate diagnosis and treatment to prevent poor clinical outcomes. This paper reports one case of severe flexor tendon sheath infection with poor outcomes that required ray amputation of the affected finger.

**Case presentation:**

A 35-year-old Sundanese male presented to the emergency department with right middle finger pain accompanied with swelling, blister, and blackened color 24 hours after accidental puncture by suture needle during gynecologic surgery. The patient was a resident physician of the obstetrics/gynecology department. The finger was necrotic with blisters at the proximal phalanx of the palmar aspect. Both the palmar and the dorsal aspects of the hand were swollen and inflamed, with firmer swelling on the dorsal part. The necrotic area had extended to the middle phalanx. The patient has been diagnosed with flexor tendon sheath infection with compartment syndrome. Immediate surgical debridement and fasciotomy with shoelace technique at the distal interphalangeal joint were performed. On the initial presentation, erythrocyte sedimentation rate, white blood cell count, and C-reactive protein were elevated. Smear culture was negative. A clear boundary of necrosis at the level of the middle phalanx of the right middle finger was found; subsequently, disarticulation at the level of the distal phalanx was performed. A ray amputation was performed to preserve the hand’s function for performing surgeries in the future.

**Conclusion:**

Prompt diagnosis and treatment of flexor tendon sheath infection are required to prevent complications. Progressive inflammation around infected soft tissue due to untreated tenosynovitis may lead to poor outcomes and may lead to the amputation of the affected finger. This condition may occur even in medical professionals; as such, awareness for proper protection during any medical procedure and prompt treatment-seeking are encouraged.

## Introduction

The most common mechanism of flexor tendon sheath infection is direct inoculation from trauma, laceration, or bite. Less frequently, contiguous spread from a local infection, such as a felon or paronychia, or hematogenous spread may occur. The avascular nature of the tendon sheath has the disadvantage of limiting the host immune system response toward bacterial invasion and proliferation.

Flexor tendon sheath infection is uncommon, but it is a true orthopedic emergency. Any cuts, scratches, or puncture wounds at hand might be underdiagnosed by a medical worker or can be easily mistaken for other infectious states such as cellulitis or septic arthritis. The presence and timing of seeding trauma can be helpful in guiding the management of the infection. Flexor tendon sheath infection is diagnosed through physical examination. Kanavel cardinal signs of flexor tendon sheath infection consisted of four signs: fusiform swelling, mild digital flexion posture, pain with passive extension, and tenderness along the flexor tendon sheath. Additional supporting examinations, particularly of inflammatory markers such as white blood cell count (WBC), erythrocyte sedimentation rate (ESR), and C-reactive protein (CRP), are performed to assess the state of inflammation [1–4].

*Staphylococcus aureus* is the most prevalent causative organism in cases of flexor tendon sheath infections, found in 40–75% of cases. Methicillin-resistant *S. aureus* (MRSA) is also relatively prevalent in such infections, found in 29% of cases in several case series. Other commonly isolated bacteria include *S. epidermidis*, β-hemolytic *Streptococcus* species, and *Pseudomonas aeruginosa*. Infections with mixed flora or Gram-negative rods are also common in immunocompromised patients. Presumptive treatment should include broad-spectrum antibiotics to treat both Gram-positive cocci and Gram-negative rods [4, 5].

Rapid assessment and diagnosis are imperative to prevent both short-term and long-term sequelae. Failure to provide prompt diagnosis and treatment may result in complications in the short and long term, such as stiffness, loss of motion across the interphalangeal joints, deformity with soft-tissue loss, osteomyelitis, or spread of infection with resultant amputation [6]. We present the case of a 35-year-old resident physician diagnosed with severe flexor tendon sheath of the right middle finger and managed by ray amputation.

## Case presentation

**Fig. 1:**
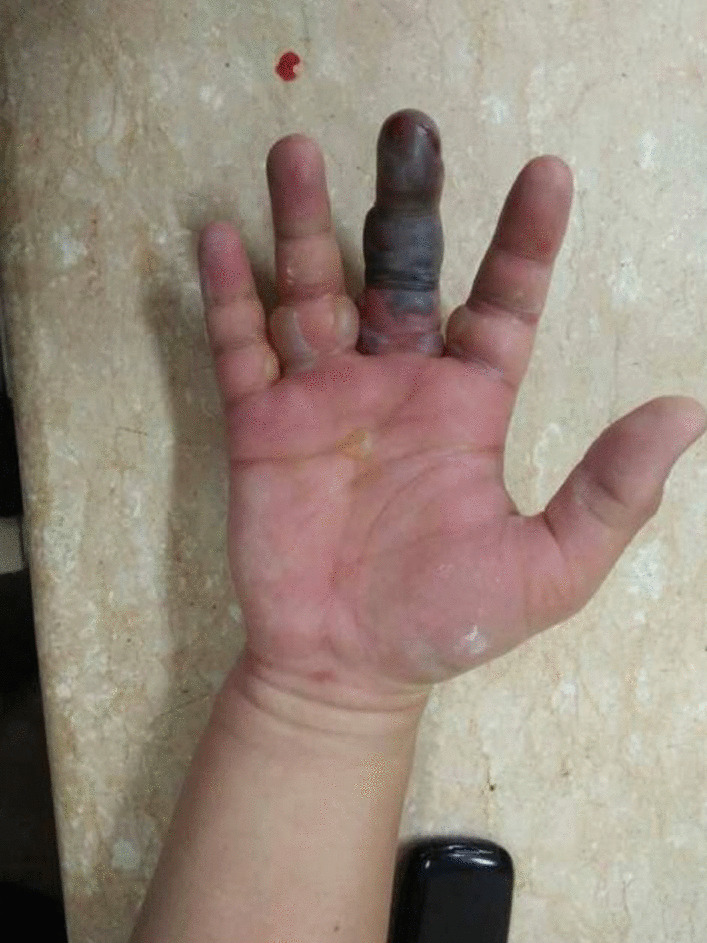
Swelling, blackening, and swelling of the hand, in dorsal and palmar aspect

**Fig. 2:**
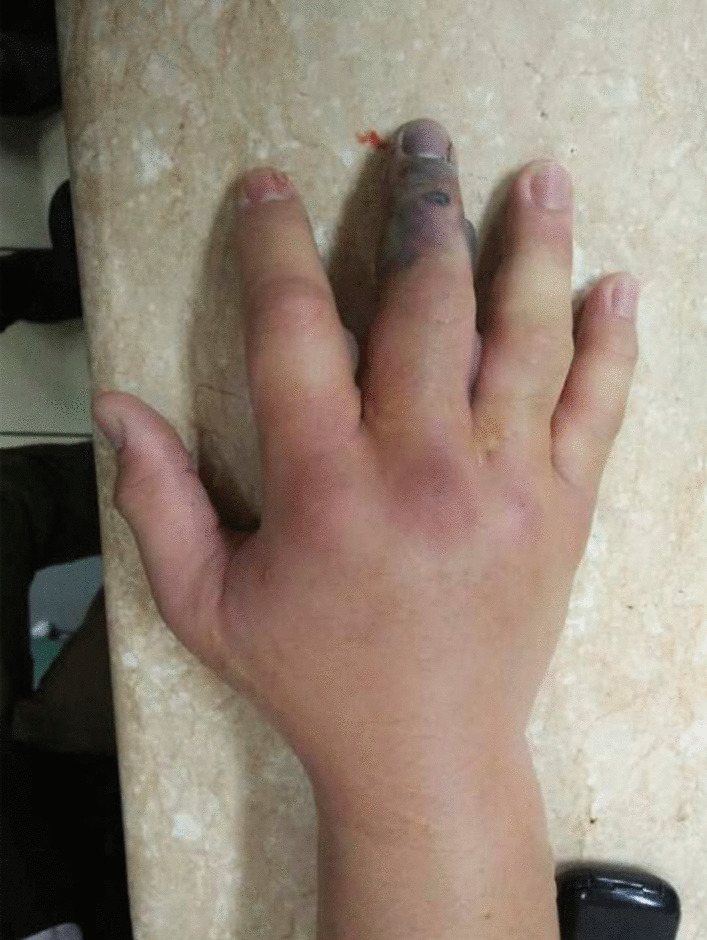
Swelling, blackening, and swelling of the hand, in dorsal and palmar aspect

A 35-year-old right-hand-dominant Sundanese male (resident physician) presented to the emergency department (ED) with the chief complaint of pain, swelling, blisters, and blackening at the right middle finger 24 hours after he had accidentally pricked the palmar aspect base distal phalanx of the right middle finger by suture needle using Chromic 1 during gynecological surgery suturing of the uterus at a rural hospital. He denied any allergies, history of sexually transmitted infections, smoking, and drinking, and had had no surgeries in the past. He had been administered broad-spectrum antibiotic (ceftriaxone 2 g) intravenously following the suture, with no improvement. Untreated, such symptoms rapidly destroy the gliding mechanism, causing adhesion formation and giving rise to necrosis. Afterward, the patient began to experience pain and swelling at the injured finger, later over his entire hand and wrist area. The middle finger had blisters and started to blacken (Figs. 1 and 2).

On physical examination, the initial wound was nowhere to be found. The middle finger had necrosis and blisters at the level of proximal phalanx of the palmar aspect. There was swelling and redness at both the palmar and dorsal aspect of the hand; the latter had tensed swelling. There were blisters and fusiform swelling at the index, middle, and ring fingers with the position of slight flexion. Passive extension and palpation of the middle finger caused severe pain. Consciousness and vital signs of the patient were within normal limits. No visible edema was found on the forearm. Laboratory studies demonstrated a total leukocyte count of 9100/mm^3^, C-reactive protein level of < 0.5 mg/L, erythrocyte sedimentation rate of 42 mm per hour, D-dimer of 0.24 µg/ml, and international normalized ratio (INR) of 1.30. On radiographic examination, there was no evidence of phalangeal fracture, bony involvement, or foreign body. He was treated empirically with ceftriaxone.

**Fig. 3:**
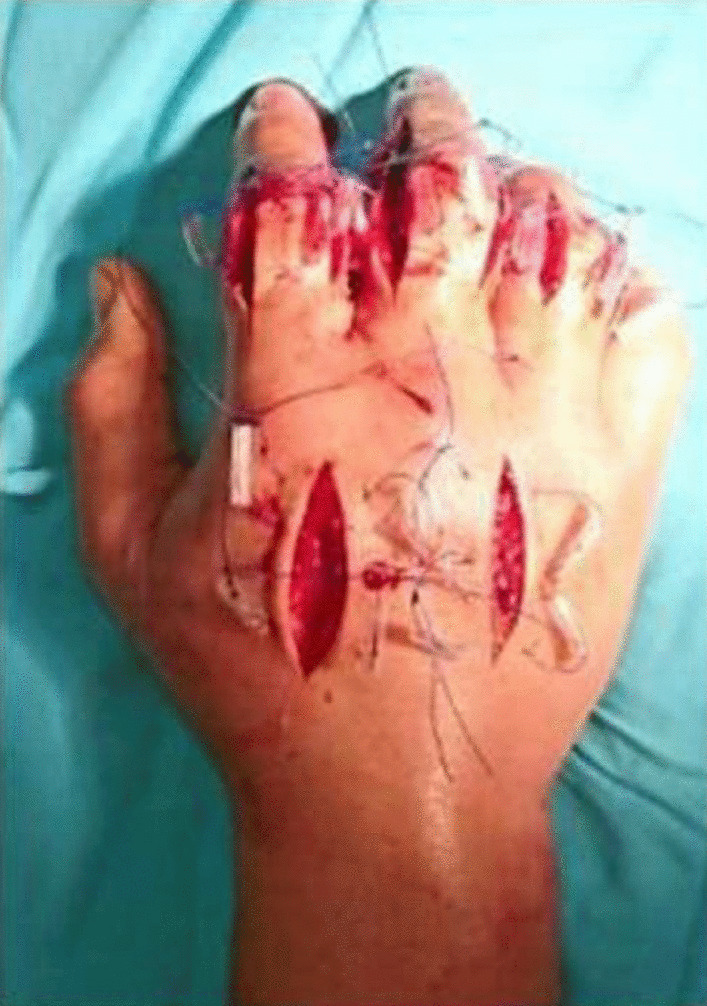
Condition after fasciotomy at dorsal aspect and finger

**Fig. 4:**
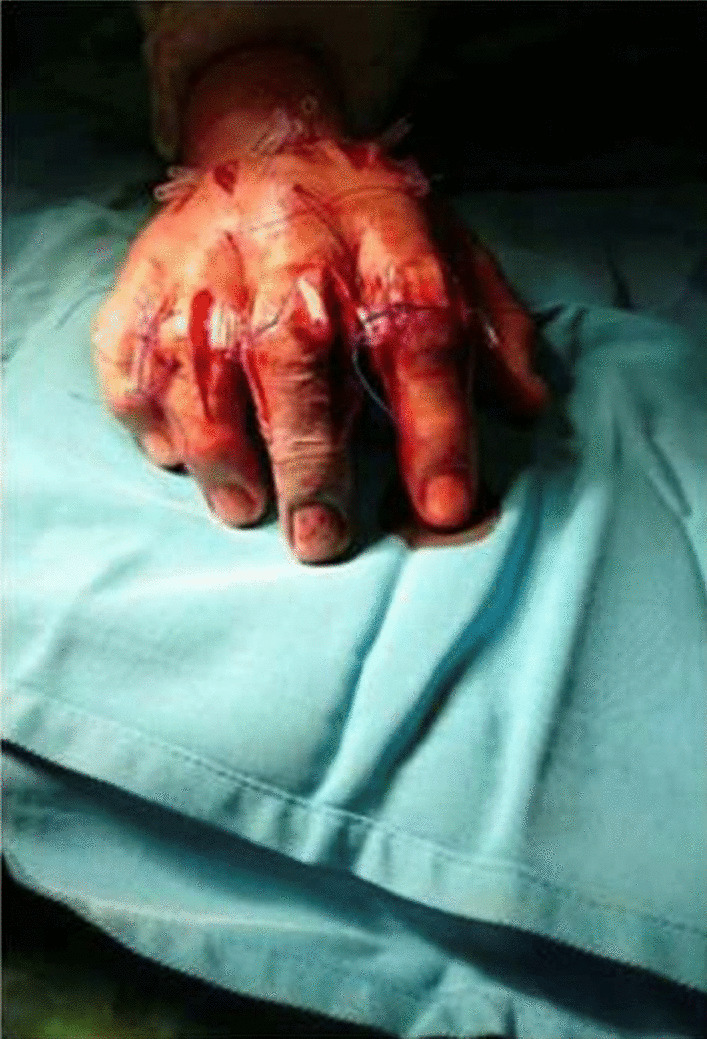
Condition after fasciotomy at dorsal aspect and finger

**Fig. 5:**
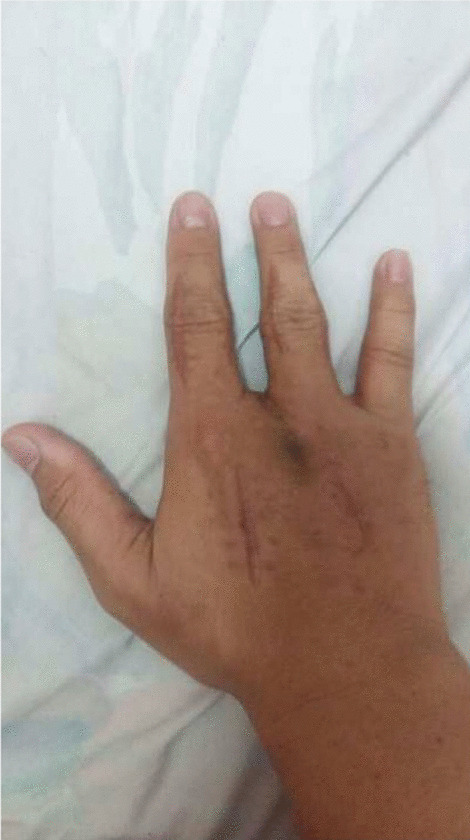
Condition after ray amputation of the middle finger

**Fig. 6:**
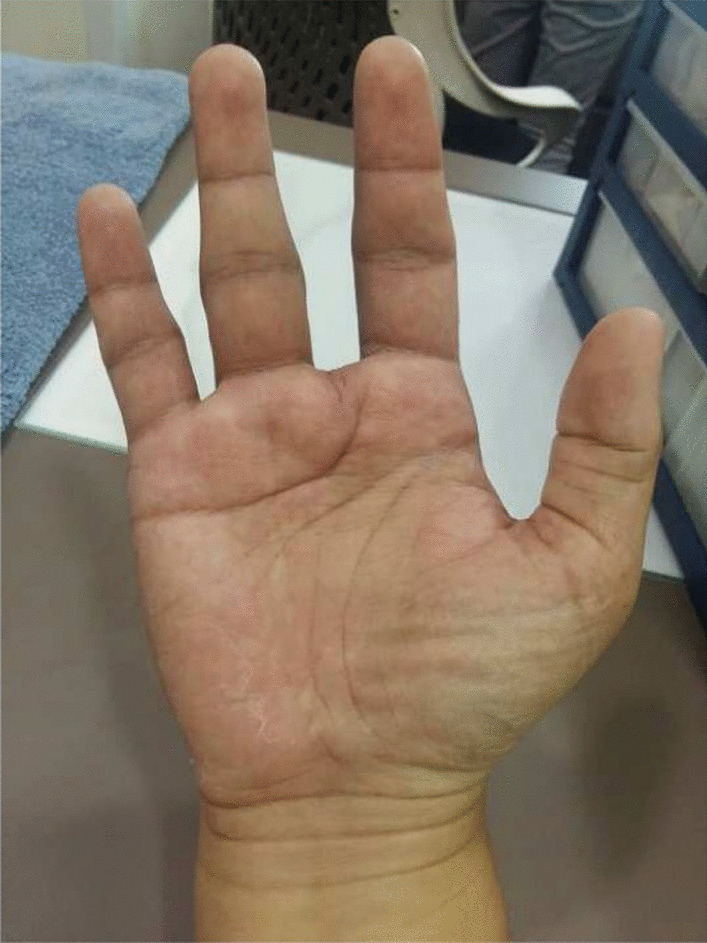
Condition after ray amputation of the middle finger

The patient was diagnosed with compartment syndrome due to a flexor tendon sheath infection stage III. Emergency surgical debridement and fasciotomy were performed. Multiple incisions were made at the dorsal aspect of the hand with two longitudinal incisions over the second and fourth metacarpals and at ulnar and radial sides of the second to fifth fingers, opening the flexor tendon sheath. Minimal purulent discharge was revealed, and cultures for aerobic, anaerobic, acid-fast bacilli, and fungus were taken. The wound was irrigated with saline and left open with a modified shoelace suture for gradual closure (Figs. 3 and 4). Follow-ups were performed daily (1–10 days) to assess the wound healing. The smear culture collected from the wound had tested negative. Afterward, the antimicrobial was subsequently changed to meropenem 1 g twice daily. On the 10th day of follow-up, the swelling was decreased. On the 20th day of follow-up, re-debridement and the primary suture were performed to evaluate the wound healing. A clear boundary of necrosis at the level of the middle phalanx of the right middle finger was found; subsequently, disarticulation at the level of the distal phalanx was performed. At the latest follow-up, 1 month after the initial surgery, examination of the affected distal phalanx was within normal limits, and there was no evidence of infection, inflammation, or swelling (Figs. 5 and 6).

**Fig. 7:**
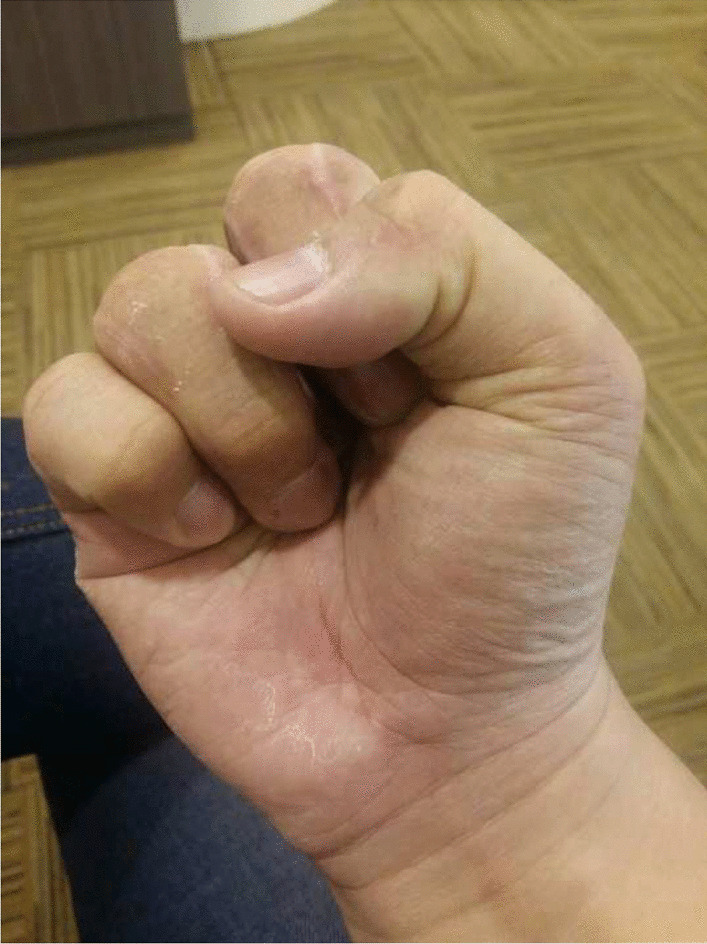
No limitation function of hand

To avoid the gap at the middle finger, the patient agreed to perform a ray amputation. At 2 years of follow-up, the patient performed physical examination and surgery without any significant difficulty (Fig. 7).

## Discussion

Flexor tendon sheath infection is predominantly a clinical diagnosis. White blood cell count, erythrocyte sedimentation rate, and C-reactive protein level can aid in diagnosis. The laboratory tests, however, were limited owing to their low negative predictive value and specificity [7]. In this case, the gynecology surgery was performed at the rural hospital. Arguably, owing to the lack of facilities for promptly treating the injury and differing sanitary standards in the operating theater of the rural hospital, the patient had presented to our ED with necrosis of the affected finger. Broad-spectrum antibiotics as empirical treatment had been reportedly given prior to the presentation on our ED. However, the antimicrobial treatment was not sufficient for halting the inflammation and subsequent swelling of the affected finger, leading to tissue ischemia and its subsequent necrosis.

Flexor tendon sheath in each finger terminates at the bony insertion of flexor digitorum profundus tendon, but there is considerable variation in the pattern of communication between the flexor tendon sheaths and bursae of the hand, whichs explain why infection in one finger can lead to direct infection of the sheath depends on individual sheath connection. A cadaver study by Fussey *et al*. [8] revealed no observed cases of communication between the middle finger tendon sheath and the ulnar bursa, ring finger and little finger sheaths, and the radial bursa, while Scheldrup [9] in 1951 observed that only 4% of middle finger flexor tendon sheath is connected to the bursae of the hand. Increased pressure in the sheath may result in the spread of infection into neighboring bursae and fascial spaces within the hand.

There were limited numbers of reports of techniques to provide decompression of the flexor tendon sheath. Potential compartments are present in the finger owing to the presence of fascial bands around the neurovascular bundles, including Grayson’s and Cleland’s ligaments. Chapman [10] preferred a Bruner zigzag incision along the volar aspect of the finger, exposing A5 and A1 pulleys. In this case, incisions were made along the ulnar and radial side of the middle and proximal phalanx of the second to fifth fingers, and optimal drainage of purulent discharges was achieved.

Management of flexor tenosynovitis has evolved to include prompt administration of intravenous antibiotics followed by surgical methods to promptly and immediately provide decompression and irrigation of the sheath. If no improvement or worsening of symptoms are seen within 12–24 hours after initiating nonsurgical treatment, the patient should undergo surgical irrigation and debridement. Ideally, empirical antimicrobial treatment should be initiated for common Gram-positive organisms, including *Staphylococcus* (MRSA) and *Streptococcus* sp. On the other hand, there is a risk of negative culture results due to prior antibiotic treatment or a vigorous immune response. The duration of antibiotic treatment depends on the patient’s clinical response; intravenous antibiotics are often followed by oral antibiotics. In recalcitrant cases, intravenous antibiotics are continued with periodical wound irrigation and debridement [4, 5].

Open irrigation and debridement procedures were originally indicated for surgical management in atypical infection cases. We prefer to close all wounds with minimal tension on the skin, and we leave space between each stitch to allow for continued drainage and to encourage a tension-free healing environment for the tissue [1–5].

Despite the aggressive management of flexor tenosynovitis, amputation was often necessary for patients with delayed presentation. Risk factors associated with an increased risk of amputation in patients with flexor tenosynovitis are (1) older age (> 43 years), (2) diabetes mellitus, peripheral vascular disease, or renal failure, (3) presence of subcutaneous purulence, (4) signs of digital ischemia at presentation, and (5) presence of multiple causative organisms [5, 6]. In our case, digital ischemia was found during the presentation. The necrotic tissue on the affected finger was found during follow-up. To preserve the other parts of the finger from possibly further affecting the fine motor skills of the patient, a ray amputation was performed.

This case report describes one of the more uncommon causes of iatrogenic flexor tendon sheath infection. Limitations include the lack of long-term follow-up on the patient after his discharge from the hospital. While the patient reportedly was able to perform surgeries after the ray amputation, the long-term effects of such amputations on occupations that require fine motor skills on the affected hand are yet to be evaluated. Further studies assessing the risk of iatrogenic tenosynovitis, particularly in surgical staff, may be required.

## Conclusion

Flexor tendon sheath infection is difficult to diagnose through clinical and microbiological methods. Flexor tendon sheath infection is a rare infection that can be easily mistaken for other infectious states such as cellulitis or septic arthritis. Therefore, the diagnosis of flexor tenosynovitis is crucial for its early proper treatment. Progressive inflammatory change in the infected soft tissue is the most common clinical course of untreated tenosynovitis. Therefore, the diagnosis of flexor tendon sheath infection is crucial for its early proper treatment such as tenosynovectomy and proper antibiotics administration. Poor outcomes like the presented case include amputation of the affected digit. Our report of complications is intended to increase awareness among residents, physicians, surgeons, and medical staff who were injured by any medical device.

## Data Availability

Consent for publication of raw data was not obtained, and the dataset could in theory pose a threat to confidentiality because the patient was also a doctor; thus, the authors respected him by not sharing the raw data with the public.
